# Harmonized definition of occupational burnout: A systematic review, semantic analysis, and Delphi consensus in 29 countries

**DOI:** 10.5271/sjweh.3935

**Published:** 2021-03-01

**Authors:** Irina Guseva Canu, Sandy Carla Marca, Francesca Dell’Oro, Ádám Balázs, Enrico Bergamaschi, Christine Besse, Renzo Bianchi, Jovanka Bislimovska, Adrijana Koscec Bjelajac, Merete Bugge, Carmen Iliana Busneag, Çiğdem Çağlayan, Mariana Cernițanu, Cristiana Costa Pereira, Nataša Dernovšček Hafner, Nadia Droz, Maija Eglite, Lode Godderis, Harald Gündel, Jari J Hakanen, Raluca Maria Iordache, Imane Khireddine-Medouni, Sibel Kiran, Francesca Larese-Filon, Catherine Lazor-Blanchet, Patrick Légeron, Tom Loney, Nicole Majery, Eda Merisalu, Ingrid Sivesind Mehlum, Laurent Michaud, Dragan Mijakoski, Jordan Minov, Alberto Modenese, Marija Molan, Henk van der Molen, Evangelia Nena, Dusan Nolimal, Marina Otelea, Elisabeta Pletea, Nurka Pranjic, David Rebergen, Jelena Reste, Eva Schernhammer, Anny Wahlen

**Affiliations:** Center for Primary Care and Public Health (Unisanté), Faculty of Biology and Medicine, University of Lausanne, Switzerland; Faculty of Arts, University of Lausanne, Switzerland; Center for Hellenic Studies, Harvard University, USA; Division of Occupational Health, Department of Preventive Medicine, Faculty of Public Health, University of Debrecen, Hungary; Department of Public Health Sciences and Pediatrics, University of Turin, Italy; Department of Psychiatry, Lausanne University Hospital (CHUV), Prilly, Switzerland; Institute of Work and Organizational Psychology, University of Neuchâtel, Neuchâtel, Switzerland; Institute of Occupational Health of RNM, WHO CC, Skopje, RN Macedonia; Institute for Medical Research and Occupational Health, Zagreb, Croatia; National Institute of Occupational Health, Oslo, Norway; National Romanian Television, Occupational Health Department, Bucharest, Romania; Department of Public Health, Faculty of Medicine, Kocaeli University, Kocaeli, Turkey; Management and Psychology department, Nicolae Testemitanu State University of Medicine and Pharmacy, Chisinau, Republic of Moldova; Environmental Health Department, National Institute of Health, Porto, Portugal; Epidemiology Unit, Institute of Public Health, University of Porto, Porto, Portugal; Clinical Institute of Occupational, Traffic, and Sports Medicine, University Medical Centre, Ljubljana, Slovenia; Occupational Health Unit, Service of Hospital Preventive Medicine, Lausanne University Hospital (CHUV), Lausanne, Switzerland; Department of Occupational and Environmental Medicine, Rīga Stradiņs University, Riga, Latvia; Department of Primary Care and Public Health, University of Leuven, Belgium; Clinic for Psychosomatic Medicine and Psychotherapy, University of Ulm, Germany; Work Careers and Workability Department, Finnish Institute of Occupational Health, Helsinki, Finland; Alexandru Darabont National Research and Development Institute for Occupational Safety and Health, Bucharest, Romania; Division of Non-Communicable Diseases and Trauma, Public Health France, Saint-Maurice, France; Institute of Public Health, Department of Occupational Health and Safety, Hacettepe University, Turkey; Unit of Occupational Medicine, University of Trieste, Italy; Department of Psychiatry, Sainte-Anne Hospital, Medical Faculty University, Paris, France; College of Medicine, Mohammed Bin Rashid University of Medicine and Health Sciences, Dubai, United Arab Emirates; Multisectoral Occupational Health Service, Luxembourg, Luxembourg; Estonian University of Life Sciences, Tartu, Estonia; Psychiatric Liaison Service, Lausanne University Hospital, Lausanne, Switzerland; Institute of Occupational Health of RNM, WHO CC, Skopje, RN Macedonia; Department of Biomedical, Metabolic and Neural Sciences, University of Modena & Reggio Emilia, Italy; Department of Occupational Medicine, Medical Faculty University of Tuzla, Bosnia and Herzegovina; Coronel Institute of Occupational Health, Netherlands Center for Occupational Diseases, Amsterdam Public Health Research Institute, The Netherlands; Medical School, Democritus University of Thrace, Alexandroupolis, Greece; National Institute of Public Health of Slovenia, Ljubljana, Slovenia; Carol Davila University of Medicine and Pharmacy, Bucharest, Romania; Department of Occupational Medicine, Medical Faculty University of Tuzla, Bosnia and Herzegovina; Occupational and Health Psychologist. Shared Ambition, People management, Amersfoort, The Netherlands; Institute of Occupational Safety and Environmental Health, Riga Stradiņš University, Latvia; Department of Epidemiology, Center for Public Health, Medical University of Vienna, Vienna, Austria; Swiss Association of Work & Organization Psychologists, Switzerland

**Keywords:** epidemiology, exhaustion, job stress, occupational health

## Abstract

**Objective:**

A consensual definition of occupational burnout is currently lacking. We aimed to harmonize the definition of occupational burnout as a health outcome in medical research and reach a consensus on this definition within the Network on the Coordination and Harmonisation of European Occupational Cohorts (OMEGA-NET).

**Methods:**

First, we performed a systematic review in MEDLINE, PsycINFO and Embase (January 1990 to August 2018) and a semantic analysis of the available definitions. We used the definitions of burnout and burnout-related concepts from the Systematized Nomenclature of Medicine Clinical Terms (SNOMED-CT) to formulate a consistent harmonized definition of the concept. Second, we sought to obtain the Delphi consensus on the proposed definition.

**Results:**

We identified 88 unique definitions of burnout and assigned each of them to 1 of the 11 original definitions. The semantic analysis yielded a first proposal, further reformulated according to SNOMED-CT and the panelists’ comments as follows: “*In a worker, occupational burnout* or *occupational physical AND emotional exhaustion state* *is an exhaustion due to prolonged exposure to work-related problems*”. A panel of 50 experts (researchers and healthcare professionals with an interest for occupational burnout) reached consensus on this proposal at the second round of the Delphi, with 82% of experts agreeing on it.

**Conclusion:**

This study resulted in a harmonized definition of occupational burnout approved by experts from 29 countries within OMEGA-NET. Future research should address the reproducibility of the Delphi consensus in a larger panel of experts, representing more countries, and examine the practicability of the definition.

Despite more than half a century of research on occupational burnout, little is known about its prevalence, etiology, treatment, or prevention. The lack of consensus on the nature of burnout has led to a proliferation of definitions and measures of the construct (1). This state of affairs has precluded a reliable estimation of its incidence and prevalence and has negatively affected the quality of research on this outcome. In the context of increasing burnout complaints (2–5) and recognition of incapacity for work due to mental ill-health (6), the need for a harmonized definition of this concept seems urgent.

A definition standardizes and regulates how a particular term should be used, ie, it is a sentence that fixes and establishes both the meaning of an expression and the syntax of its use (7). Therefore, definitions have an instrumental value as they help to systematize knowledge (8). Moreover, when introducing a new term into a vocabulary, definitions enhance its formal-expressive power. Controlled vocabulary or terminology is designed by a group of experts and only contains authorized technical terms of a specific field (8). In the field of medicine, the Systematized Nomenclature of Medicine Clinical Terms (SNOMED-CT) is the most comprehensive and reliable terminology (9, 10). For example, it contains the term ’burnout’ and its definition. Nonetheless, most professionals are unaware of its existence, instead referring to the International Statistical Classification of Diseases and Related Health Problems (ICD) (11). Yet, the ICD is a coding system aimed at statistically classifying medical information. ICD is not a nomenclature of medical terms, aiming to provide their definition. This explains why, the entity “*burn-out*” was introduced in the 10^th^ revision of ICD (ICD-10) without any definition (12). Conversely, the somewhat arbitrary definition of burnout provided in ICD-11 appears misleading. However neither its changed ICD code (from Z73 to QD85) nor transfer from the subsection “Problems related to life management difficulty” to the subsection “Problems related to employment or unemployment” would justify the sudden need for a definition of burnout in the ICD. Instead, the introduction of a new term (eg, “work-related burnout”) along with an appropriate definition may be warranted, given that such a term has not yet been defined in any official medical terminology.

In controlled terminology, a definition is a sentence suggesting that a new term (the *definiendum*) should be considered as synonymous with another, already known term or expression (the *definiens*) (7). The only exception is the so-called “*ostensive* definition”, where the term is interpreted by pointing to an object and naming it (eg, “You will be called XYZ”). In fact, the term burnout was originally introduced using an ostensive definition (13), and only later explained by Freudenberger (14) and many others. All of them are explanations, not definitions for a controlled vocabulary. Some are so-called “meaning explanations”, attempting to explore what people understand by a term such as burnout, and others are descriptions, enumerating properties and attributes of burnout. All belong to the natural language vocabulary.

Given this situation, we aimed to (1) formulate a harmonized definition of the concept of occupational burnout for its introduction in the medical vocabulary and (2) reach a consensus on the definition and most appropriate term to designate this concept within the the Network on the Coordination and Harmonisation of European Occupational Cohorts OMEGA-NET, part of the EU European Cooperation in Science and Technology (COST) Action (15, 16).

## Methods

We conducted this research in two parts. First, we performed a systematic review of all existing definitions of occupational burnout and a semantic analysis of the 11 original definitions. We used Systematized Nomenclature of Medicine Clinical Terms (SNOMED-CT) definitions of burnout and burnout-related concepts to propose the terms and a definition of the concept. Secondly, we sought to obtain consensus on our proposal using the Delphi technique (17, 18).

### Systematic review and semantic analysis

*Search strategy and selection criteria*. The search was conducted within the context of a broader systematic review, aimed at addressing all causative predictors of burnout in workers (PROSPERO CRD42018105901) (19). We searched the literature published between January 1990 and August 2018 on MEDLINE, PsycINFO and EMBASE. [The complete search strings applied for each database are available at the Unisanté data repository (DOI: 10.16909/DATASET/22).] We validated this search strategy by achieving exhaustiveness of the studies included in the latest systematic review on occupational burnout (2). In addition, we checked the reference lists of all retrieved articles and reviews to look for additional studies, which could be included.

We included original research focused on workers, published in European languages between 1990–2018 in peer-reviewed journals. Among them, we selected studies which (i) examined the relationship between exposure to any kind of factors (eg, occupational, organization, individual) and the onset of burnout; (ii) used a longitudinal design, (iii) assessed exposure before the onset of burnout, and (iv) had a minimum of 50 participants per exposed group. When multiple publications described the same study, we included the publication with the most complete reporting of study results. We conducted a double screening of relevant studies: the first screening was based on the title and abstract of all publications identified through the literature search. All studies which met the inclusion criteria, or for which it was not possible to check these criteria, were included in the second screening, which was based on reading the full text. The literature corpus was equally allocated between the 14 OMEGA-NET reviewers. In parallel, the second reviewer independently read all the studies. Therefore, two independent reviewers conducted both screenings. A third reviewer helped resolving disagreements.

For this study, OMEGA-NET reviewers extracted for each study: the reference, year of publication, definition of burnout as formulated by the authors (ie, used definition) and the source(s) of this definition (ie, referenced definition(s)) using a standardized data extraction form (MS Excel). The first and second authors double-checked all extracted data.

### Semantic analyses and definition proposal

The referenced and used definitions were split between original definitions (ie, a definition published for the first time by the authors to introduce their theoretical concept) and secondary definitions [ie, a definition by the same author(s), based on the same concept as the original definition, but formulated using a different wording (synonyms)]. All definitions (original and secondary) constituted the corpus of our semantic research. However, the analytical sub-corpus only included the original definitions and two additional definitions published after the completion of the literature search (20, 21).

We conducted the semantic analysis in three phases: In phase 1, we examined the concepts and their expressions in terms of hyponymy and hyperonymy, corresponding to the lower (more specific) and upper (more general) levels in the concept’s semantic hierarchy, respectively. We selected hyponyms and hyperonyms occurring/recurring in at least three different definitions. The choice of this number is arbitrary, but justified, as the choice of a low number prevents the loss of potentially interesting information. We considered that the (hyponymic or hyperonymic) terms that appeared only once or twice were too specific and hence not worth taking into account. Definitions of burnout were all structured in a heterogeneous way and described in the form of (i) a list of simple terms (eg, stress, boredom, frustration), (ii) a list of nominalizations with some specifications (eg, “feeling of exhaustion and fatigue, being unable to shake a lingering cold, suffering from frequent headaches and gastrointestinal disturbances”), but also (iii) in a more discursive way (eg, “They lose all concern, all emotional feelings, for the persons they work with…”). We also considered discursive descriptions as lists of elements, so that, for example, “they lose all concern” can be interpreted and evaluated as “loss of all concern”. In this way, single terms and multi-word expressions can be considered as isolated semantic elements, independently from a specific and actualizing syntactic context.

In phase 2, we reorganized the results of phase 1 into the ideal structure for the medical description of burnout as a syndrome, ie, a multi-level conceptual framework based on symptoms. To enhance the precision of the level to which symptoms should be attributed, we excluded all information about the context of burnout development and the specific population prone to burnout, which were in the definitions.

In phase 3, we calculated the effective presence of each element on each level, in each definition. We deduced a semantic proposal of a definition of occupational burnout based on shared elements (ie, the elements that occurred in more than half or ≥7 out of the 13 definitions of the analytical sub corpus).

Furthermore, we consulted the last release (July 2019) of SNOMED-CT International Edition for the terms “burnout”, “exhaustion”, and “occupation(al)” and extracted their definitions and the definitions of their hyperonyms and hyponyms. We summarized the extracted information and formulated a definition proposal based on SNOMED-CT’s terminology, following the fundamentals of medical concept formation (7).

### Consensus search through the Delphi process

We considered as experts all members of OMEGA-NET and external experienced health practitioners with ≥10 years of practice and knowledge of occupational burnout. We used purposive sampling among OMEGA-NET members and snowball sampling with the external health practitioners. The latter method was implemented through the national focal points of the European Agency for Safety and Health at Work (EU-OSHA) (22) and OMEGA-NET members, who we asked to identify at least one health practitioner in each of the 33 OMEGA-NET participating countries (16). We used this approach previously (6) and found it effective. The working language was English. We sent an invitation by e-mail describing the Delphi protocol and time-schedule. This initial e-mail helped to establish a relationship with and verify the e-mail addresses of experts. It also provided the denominator to calculate the response rate.

We *a priori* defined the consensus valid if at least 75% of participants rated the definition ≥7 on a 9-point Likert scale (23). We provided to the expert panel a synthesis of the evidence resulting from the systematic literature review and semantic analysis, which were conducted prior to the consensus process. Therefore, panelists received all pertinent information enabling their evidence-based decision-making (24). We also sent them detailed instructions of the process.

We restricted the process to two rounds as more rounds would have increased the panel’s attrition (25). In the first round, we used a questionnaire with a choice of two terms for designating the concept of occupational burnout and the proposal of its definition. Panelists were asked to rate their agreement with the definition using a 9-item Likert scale. Panelists were also asked, in an open-ended question, to explain their rating and express the reasons of their agreement/disagreement with the definition statement. They were also encouraged to share their comments and/or suggestions for amendments on the proposed definition. We sent two reminders to non-responders by e-mail. We collated the responses of the first-round questionnaire and used them to create the second-round questionnaire, which presented a slightly revised statement of definition. Panelists also received a document summarizing the first round rating statistics along with a selection of free-text responses to represent the breadth of opinion of participants. Experts reconsidered their previous opinion and rerated their degree of agreement with the new proposed definition. The reratings were summarized and assessed for degree of consensus. At the end of the process, all participants were provided the results.

## Results

**Figure 1:**
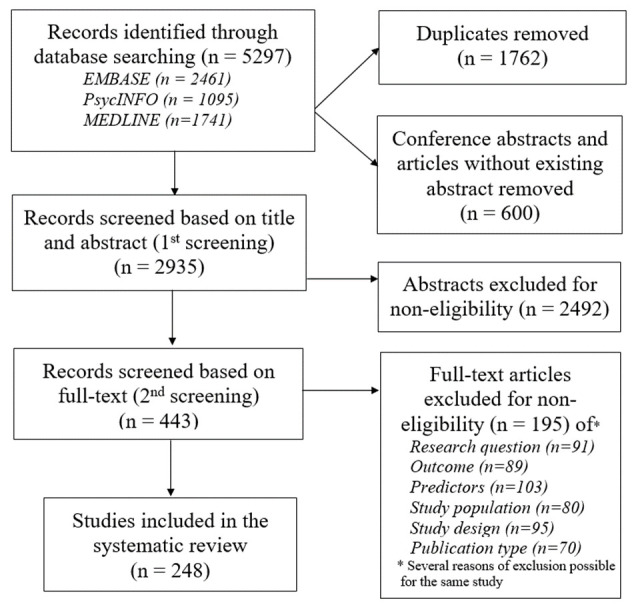
Flow diagram of study selection

**Figure 2:**
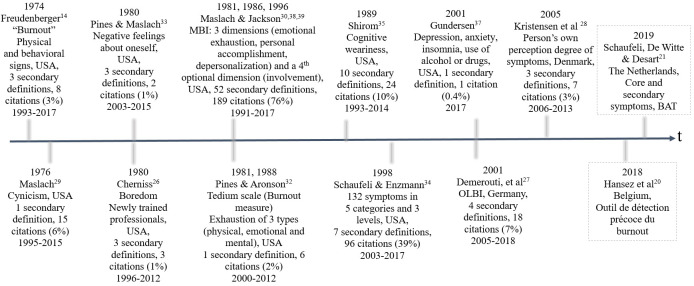
Chronology of original definitions of occupational burnout. For each referenced original definition, the year of the first and updated publication, the first author’s name, main features, and the country of publication are reported along with the number of the corresponding secondary definitions, the frequency [N(%)]* and the timespan of their citations as quantified in the frame of the systematic review of 248 longitudinal studies on occupational burnout. The two definitions identified after the end of the systematic literature search (August 2018) were added and shown framed in dotted lines. *A single article can cite more than one definition.

The systematic literature search produced 5297 items. After the first and second screenings of 2935 abstracts and 443 articles, respectively, 248 studies met the inclusion criteria (figure 1). After comparative analysis of the 248 extracted definitions, we grouped together those with very similar content. This resulted in 88 distinct definitions. Most definitions were ranked as secondary, referring to 1 of the 11 original definitions (14, 26–37). The references of the 248 studies, 88 secondary definitions and their indexation to 11 original definitions are available upon request via Unisanté data repository (DOI: 10.16909/DATASET/22). Table 1 presents the statements of all original definitions, their comparative features and the theoretical ground of their development. Figure 2 presents these original definitions in a chronological way, along with the number of their secondary definitions, the frequency and the timespan of their citations in the studies included in the systematic review. The second revision of Maslach & Jackson’s definition (30, 38, 39) was the fourth to be published but appears as the most commonly used definition (76%) for assessing burnout as a health outcome in workers. However, a two-fold revision of this definition and the subsequent publication of nine other new definitions attests that Maslach & Jackson’s definition has no unanimous acceptance. The second most common definition was that of Shaufeli & Enzman (34) (39% of citations). While Maslach & Jackson’s definition describes burnout in terms of three core dimensions (emotional exhaustion, depersonalization and personal accomplishment), which can be measured by a self-administrated scale (the Maslach Burnout Inventory or MBI), Shaufeli & Enzman’s definition is largely descriptive, listing 132 symptoms, which they considered likely of burnout cases (34). Considering the chronology of the original definitions, a comparative analysis revealed some minor and inconsistent changes in the theoretical models on which the identified definitions were based and an increasing complexification of the definition content (table 1).

**Table 1:** Comparative analysis of the original definitions in terms of at-risk population, symptoms and theoretical model or tool related.

Author,year (ref)	Definition	Comparative features
Freudenberger, 1974 (14)	Physical (feeling of exhaustion and fatigue, being unable to shake a lingering cold, suffering from frequent headaches and gastrointestinal disturbances, sleeplessness and shortness of breath) and behavioral (a staff member’s quickness to anger and his instantaneous irritation and frustration responses are the signs) signs of burnout.	Population: “The dedicated and the committed” people Model: Transposition into words of the burnout concept
Maslach, 1976 (29)	People who work intensively with others […] are often unable to cope with this continual emotional stress and burnout occurs. They lose all concern, all emotional feeling, for the persons they work with and come to treat them in detached or even dehumanized ways. […] The worker’s feelings about people often show a shift toward the cynical or negative. […] Burnout often leads to a deterioration of physical well-being. The professional becomes exhausted, is frequently sick and may be beset by insomnia, ulcers and migraine headaches, as well as more serious illnesses.	Population: People who work intensively with other Symptoms: Emotional stress; Loss of all concern for the persons professionals work with -> cynical feelings; Physical exhaustion Model: emotional stress -> burnout -> deterioration of physical well-being
Pines & Maslach, 1980 (33)	Burn-out is a syndrome of emotional exhaustion and cynicism that can occur among individuals who spend much of their time working closely with other people. It involves a gradual loss of concern for these other people and the development of callous and even dehumanized attitudes towards them, and it can sometimes result in negative feelings about oneself as a professional helper or care-giver. The emotional fatigue of burn-out can have detrimental effects on the individual’s job performance (as reflected in lower morale and greater absenteeism and turnover), as well as on his or her physical health (increased physical exhaustion, psychosomatic symptoms, and vulnerability to disease). Furthermore, it can seriously affect the individual’s psychological well-being and impair his or her ability to relate to people in general (and not just to the recipients of his or her professional services). Burn-out is not unique to a particular group of individuals but is found among most health and service professions where staff members are required to work intensively with people on a large-scale, continuous basis in situations that can be emotionally demanding (Freudenberger, 1977; Kafry and Pines, 1979; Maslach, 1976, 1978a, 1978b, 1979; Maslach and Jackson, 1978, 1979; Maslach and Pines, 1977, 1979; Mattingly, 1977; Pines and Kafry, 1978, 1979; Pines and Maslach, 1978; Reed, 1977).	Population: Not unique to a particular group of individuals but is found among most health and service professions where staff members arerequired to work intensively with people on a large-scale Symptoms: Negative feelings about oneself as a professional helper or care-giver Model: Working closely with people -> burnout -> detrimental effects on individual’s job performance, on physical and psychological health, and on the ability to interact with people in general
Cherniss, 1980 (26)	Professional burnout is described as a syndrome of many negative factors. These include stress, strain, boredom, self-doubt, dissatisfaction, insecurity, disappointment, and frustration. Burnout is usually experienced by some newly trained professionals who are employed in large bureaucratic public agencies, frequently’ during their first professional appointment.	Population: Newly trained professionals who are employed in large bureaucratic public agencies Symptoms: Boredom
Maslach & Jackson, 1981 (30), 1986 (38), 1996 (39)	Burnout is a syndrome of emotional exhaustion and cynicism that occurs frequently among individuals who do ‘people-work’ of some kind. A key aspect of the burnout syndrome is increased feelings of emotional exhaustion. As their emotional resources are depleted, workers feel they are no longer able to give of themselves at a psychological level. Another aspect is the development of negative, cynical attitudes and feelings about one’s clients. Such negative reactions to clients may be linked to the experience of emotional exhaustion, i.e. these two aspects of burnout appear to be somewhat related. This callous or even dehumanized perception of others can lead staff to view their clients as somehow deserving of their troubles (Ryan, 1971), and the prevalence among human service professionals of this negative attitude toward clients has been well documented (Wills, 1978). A third aspect of the burnout syndrome is the tendency to evaluate oneself negatively, particularly with regard to one’s work with clients. Workers feel unhappy about themselves and dissatisfied with their accomplishments on the job.	Tool: Development of the Maslach Burnout Inventory (MBI) with 3 dimensions and a 4th optional dimension (1.Emotional exhaustion 2.personal accomplishment 3.depersonalization 4.involvement) Comment: definition revised by the authors in 1986 and 1996
Pines &Aronson, 1981, (32)	Burnout is identical to tedium in terms of definition and symptomology but is unique to people who work with people in situations that are emotionally demanding. Tedium is the experience of physical, emotional, and mental exhaustion. It is characterized by emotional and physical depletion and by the negation of one’s self, one’s environment, one’s work, and one’s life.	Population: people who work with people in situations that are emotionally demanding Symptoms: Physical and mental exhaustion in addition to emotional exhaustion. Not only negation of one’s work, but also negation of one’s self, one’s environment and one’s life. Model: Burnout is an experience Comment: definition revised by the authors in 1988, while introducing the Burnout Measure (BM) tool with 3 dimensions (physical, emotional, and mental exhaustion)
Shirom, 1989 (35)	Individual level phenomenon. A negative emotional experience. A chronic ongoing feeling. The unique content of burnout has to do with the depletion of an individual’s energetic resources. Specifically, burnout refers to a combination of physical fatigue, emotional exhaustion, and cognitive weariness. […] There are several underlying assumptions often made by burnout researchers that need to be discarded if one accepts the core definition of burnout […] They need not, and should not be restricted to individuals whose work requires large amounts of contact with people in need of aid (Maslach & Jackson, 1984). Therefore, a theory of burnout must not allow itself to be exclusively concerned with the people occupations. Yet another assumption often made by burnout researchers (e.g. Jackson, Schwab, and Schuler, 1986) is that the term exhaustion means that the burnout syndrome is most relevant for job holders whose work is very involving. In face, most studies reported moderate negative correlations between burnout and work involvement or commitment (Farber, 1984). A third assumption found in burnout research is that it is often preceded by high levels of arousal (Maslach, 1982b; Edelwich and Brodsky, 1980). Again, this is not necessarily implied by the above core definition.	Symptoms: Cognitive weariness Comment: In 1992 the author reproduced this definition, while introducing the Shirom-Melamed Burnout Measure (SMBM), measuring physical fatigue and emotional exhaustion.
Schaufeli & Enzmann,1998 (34)	Myriad possible burnout symptoms (132 symptoms displayed on table 2) and definitions exist. Symptoms are in five clusters: affective, cognitive, physical, behavioral, and motivational. Three levels are distinguished: individual, interpersonal, organisational. Two types of definition: by symptoms and by process. Both types are complementary as the symptoms are the end-state of the process. Most common symptoms def = Maslach & Jackson 1986. […] Burnout is a persistent, negative, work-related state of mind in “normal” individuals that is primarily characterised by exhaustion, which is accompanied by distress, a sense of reduced effectiveness, decreased motivation, and the development of dysfunctional attitudes and behaviours at work. This psychological condition develops gradually but may remain unnoticed for a long time by the individual involved. It results from a misfit between intentions and reality in the job. Often burnout is self-perpetuating because of inadequate coping strategies that are associated with the syndrome. (This working definition of burnout specified its general symptomatology, its pre-conditions, as well as the domain on which it occurs. More specifically, the definition narrows down over 100 burnout symptoms to one core indicator (exhaustion) and four accompanying, general symptoms (1): distress (affective, cognitive, physical, an behavioral) (2); a sense of reduced effectiveness (3); decreased motivation (4); dysfunctional attitudes and behaviours at work. Furthermore, frustrated intentions and inadequate coping strategies play a role as preconditions in the development of burnout and the burnout process is considered to be self-perpetuating despite the fact that it may not be recognised initially. Finally, the domain is specified: the symptoms are work-related and burnout occurs in “normal” individuals who do not suffer from psychopathology).	Population: “normal” individuals who do not suffer from psychopathology Symptoms: 132 symptoms distinguished in five clusters (affective, cognitive, physical, behavioral, and motivational) and in three levels (individual, interpersonal, organizational). Those symptoms can be summarized in exhaustion, which is accompanied by distress, a sense of reduced effectiveness, decreased motivation, and the development of dysfunctional attitudes and behaviours at work. Model: Two complementary types of burnout definition exist: by process and by symptoms. The symptoms definition is the end state of the process definition. Comment: In 2000, Schaufeli & van Dierendonck produced a Dutch translation of the Maslach Burnout Inventory (MBI). It has 15 items and three subscales (emotional exhaustion, mental distance, and competence) and used Maslach & Jackson’s definition as it is, translated into Dutch.
Demerouti et al., 2001 (27)	This state, where both exhaustion and disengagement are simultaneously present, represents the burnout syndrome. According to our conceptualization, burnout represents a dichotomous and not a continuous trait, as in Maslach’s concept, where burnout can have low, medium, or high levels.	Population: not defined Symptoms: Suppression of the disengagement Model: Burnout is a dichotomous and not a continuous trait. Tool: OLdenburg Burnout Inventory (OLBI) with two dimensions (exhaustion, disengagement)
Gundersen, 2001 (37)	Burnout has many characteristics, including fatigue, exhaustion, inability to concentrate, depression, anxiety, insomnia, irritability, and sometimes increased use of alcohol or drugs. Probably the most distinct characteristic of burnout is a loss of interest in one’s work or personal life, a feeling of “just going through the motions.”	Population: not defined Symptoms: Inclusion as symptoms of previously seen as consequences of burnout: inability to concentrate, depression, anxiety, insomnia, irritability, and sometimes increased use of alcohol or drugs.
Kristensen et al, (2005) (28)	In the CBI the core of burnout is fatigue and exhaustion. […] While ‘‘the flat battery’’ remains the main metaphor for burnout, it is important to emphasize that burnout is not just fatigue or exhaustion. If this were the case we would not need the concept at all. In our understanding of the concept the additional key feature is the attribution of fatigue and exhaustion to specific domains or spheres in the person’s life. One such domain is work and a more specific domain is client work. Work-related burnout: The degree of physical and psychological fatigue and exhaustion that is perceived by the person as related to his/her work’’ […] Client-related burnout: The degree of physical and psychological fatigue and exhaustion that is perceived by the person as related to his/her work with clients. The additional key feature is the attribution of fatigue and exhaustion to specific domains or spheres in the person’s life. One such domain is work and a more specific domain is client work.	Population: people working with clients Symptoms: Physical and psychological fatigue Attribution to work/client/personal domain Model: Person’s own attribution of symptoms to personal domain, work-related domain, or client-related domain. Tool: Copenhagen Burnout Inventory (CBI) with three dimensions (personal burnout, work-related burnout, client-related burnout) Comment: Although the first publication of this definition was in 1999, most authors cited the 2005 publication, in English. The 1rt author, Dr. Kristensen, confirmed that the two definitions are identical.
Schaufeli et al, 2019 (21)	Burnout is a work-related condition that occurs in those who have worked productively and without problems for a long period to the satisfaction of themselves and others. Extreme fatigue, disruption of emotional and cognitive processes, and mental distance are the core elements of the disorder. The mental distance can be seen as a dysfunctional attempt to prevent further exhaustion. These core symptoms are accompanied by secondary symptoms, such as depressive feelings, and psychosomatic and psychological stress complaints [Free translation from Dutch]	Population: Those who have worked productively and without problems for a long period to the satisfaction of themselves and others Model: Core and secondary symptoms Tool: Burnout Assessment Tool (BAT) with four dimensions (exhaustion, mental distance, emotional disturbance, cognitive trouble).
Hansez et al, 2018 (20)	Burnout is defined as a persisting negative state of mind related to work, in « normal » individuals, characterized by exhaustion, a feeling of inefficacy, a demotivation, dysfunctional behaviors at work. [Free translation from French]	Population: “Normal” individuals Tool: Tool for early burnout detection with three kinds of symptoms (physical, cognitive and emotional, behavioral)

Phase 1 of the semantic analysis revealed an absence of homogeneity in the structure of the original burnout definitions. Indeed, they sometimes referred to symptoms but also to causes or to effects. Moreover, some definitions were very precise in their terminology while others only contained generic terms. Therefore, in phase 2 of analysis, we applied an adaptation of the structural-generative semantics approach (40–43). When all the concept elements shared in the sub-corpus were classified according to a hierarchy based on three main levels (psychological, physical and behavioral), we observed that burnout symptoms at the psychological level were more numerous than those at the physical level and the latter were more numerous than the symptoms at the behavioral level. Phase 3 enabled us to calculate the occurrence of the symptoms in the original definitions for each level and layer. Elements that occurred in ≥7 out of 13 original definitions (11 original definition plus two recent definitions) (20, 21) were retained for a shared semantic definition proposal. Table 2 summarizes the results of the semantic analysis. Further details on this analysis can be found elsewhere (Dell’Oro & Guseva Canu. From semantic decomposition of the lexicon to extra-linguistic understanding of its use in the definitions of ’burn-out’ as a work-related health condition: advantages and limits of semantic decomposition emerged from a practical application. Submitted to J Applied Linguistics.)

**Table 2:** Multi-level and multi-layer structure of a semantic definition of occupational burnout based on the concepts (reduced to hyponyms or hyperonyms) shared in the analytical sub-corpus of definitions and number of their occurrence in the original definitions at each level and layer.

	Concept occurrence among 11 definitions	Concept occurrence among 13 definitions
**Psychological level**		
Deterioration of well-being	11	13
Exhaustion	8	10
Emotional exhaustion	4	4
Mental exhaustion	1	1
Weariness	5	7
Cognitive weariness	3	
Demotivation	2	3
Inability to cope	2	2
Negative attitude	7	7
Frustration	4	4
Negative feelings about oneself	4	4
Dehumanization	3	3
Detachment distancing	5	6
Detachment towards co-workers	2	2
Detachment towards clients	1	1
**Physical level**		
Deterioration of well-being	9	11
Recovery problems	3	3
Sleep disorders	3	3
Sleepiness	1	1
Insomnia	2	2
Exhaustion	9	11
Physical exhaustion	7	9
Fatigue	4	5
**Behavioural level**		
Dysfunctional behaviours	5	6
Relational inability	3	3
Regarding clients	0	0
Regarding co-workers	1	1
Cynicism (disengagement)	2	2

The resulting shared definition of occupational burnout was as follows: “a syndrome characterized by ’deterioration of well-being’ and more precisely ’exhaustion’, ’weariness’ and ’negative attitude’ at the psychological level, and ’deterioration of well-being’ with presence of ’exhaustion’ at the physical level. It is not yet possible to specify any symptom at the behavioral level. Importantly, in 12 of the 13 definitions, burnout is explicitly related to workplace.”

Table 3 presents the terms included in the shared semantic definition as defined in SNOMED-CT. ’Burnout’ and ’physical AND emotional exhaustion state’ are both descriptors of the same concept in SNOMED-CT. However, even if ’burnout’ is an acceptable term for this concept, ’physical AND emotional exhaustion state’ is specified as the preferred term in the English language reference set of both Great Britain and the United States of America. The term ’exhaustion’ is defined as ’general problem AND/OR complaint’ and ’energy and stamina’. The term ’exhaustion due to exposure’ is hyponym of ’exhaustion’. We identified the concept of ’problems at work’ as the most relevant exposure to put in relation with ’exhaustion due to exposure’. The ’problems at work’ concept is defined as ’work and retirement-related problems’ and has 12 hyponyms including ’bullied at work’, ’discord in the workplace’, and ’stressful work schedule’. ’History taking’ is specified as a method for diagnosing problems at work. Finally, the qualifier ’occupational’ is defined as a ’modifier related to clinical specialty AND/OR occupation’ (table 3).

**Table 3:** Terms identified through the comparative and semantic analyses of burnout definitions, as defined in the Systematized Nomenclature of Medicine Clinical Terms (SNOMED-CT, July 2019 release). [SCTID=SNOMED-CT identifier]

Fully specified name SCTID	Preferred synonym	Acceptable synonyms	Type of relationship (Attribute)	Descriptor	Hyponym concepts
Physical AND emotional exhaustion state (disorder)	58535001	Physical AND emotional exhaustion state	Burnout	Is a	Anxiety disorder (disorder), 197480006	None
				Interprets	Emotion (observable entity), 285854004	
Exhaustion (finding)	60119000	Exhaustion	Washed out, Worn out	Is a	General problem AND/OR complaint (finding), 105721009	6: eg, Exhaustion - physiological (finding) including Exhaustion due to excessive exertion (finding) and Exhaustion due to exposure (finding)
				Is a	Energy and stamina finding (finding), 359752005	
				Interprets	Energy / stamina (observable entity), 359755007	
Exhaustion - physiological (finding)	242015007	Exhaustion - physiological	None	Is a	Exhaustion (finding),60119000	None
				Interprets	Energy / stamina (observable entity), 359755007	
Exhaustion due to exposure (finding)	88164008	Exhaustion due to exposure	None	Is a	Exhaustion - physiological (finding), 242015007	2: Exhaustion due to excessive exertion (finding) and Exhaustion due to exposure (finding)
				Interprets	Energy / stamina (observable entity), 359755007	
Energy / stamina (observable entity)	359755007	Energy / stamina	Energy and stamina, Observation of energy and stamina	Is a	Metabolic observable (observable entity), 364392006	5: eg, Activity tolerance (observable entity), Endurance (observable entity), Level of fatigue (observable entity)
Problems at work (finding)	266959008	Problems at work	None	Is a	Work and retirement-related problems (finding), 302122003	12: eg, Bullied at work (finding), Business worries (finding), Work maladjustment problem (finding), Stressful work schedule (finding)
				Finding method	History taking (procedure), 84100007	
				Interprets	Legal, financial, employment and socioeconomic history detail (observable entity), 302148006	
Occupational disorder (disorder)	115966001	Occupational disorder	None	Is a	Environment related disease (disorder), 8504008	14: eg, Disorder due to work-related activity accident (disorder), Effects of exposure to extreme temperature, occupational (disorder), Gulf war syndrome (disorder)
Environment related disease (disorder)	8504008	Environment- related disease	None	Is a	Disease (disorder), 64572001	7: eg, Idiopathic environmental intolerance (disorder), Industrial / Institutional / Natural environment related disease (disorder)
Disease (disorder)	64572001	Disease	Clinical disease AND/OR syndrome, Disease AND/OR syndrome present, Syndrome	Is a	Clinical finding (finding), 404684003	86, eg, Acute disease (disorder), Chronic disease (disorder)
Occupational (qualifier value)	87923000	Occupational	None	Is a	Modifier related to clinical specialty AND/OR occupation (qualifier value), 106236003	None
Occupational hazard (qualifier value)	17458004	Occupational hazard	None	Is a	Any hazardous entity (qualifier value), 21703008	22: eg, Mining of hazardous mineral (qualifier value), Specific occupational equipment/hazard (qualifier value)
History taking (procedure)	84100007	History taking, A clinically oriented interview of a patient or someone familiar with the patient	Clinical interview, History taking, health, Taking health history	Is a	Interview, history AND/OR physical examination (procedure), 108217004	13: eg, History AND physical examination (procedure), History taking, self-administered, questionnaire (procedure),
				Method	History taking - action (qualifier value), 129431000	

Considering these definitions, we proposed to introduce a new concept using two synonymous terms: ’occupational physical AND emotional exhaustion state’ (term 1) and ’occupational burnout’ (term 2). We defined it as follows: “*In a worker, occupational physical AND emotional exhaustion state or occupational burnout is an exhaustion due to exposure to problems at work*”. This proposal was submitted for experts’ approval.

Among the 100 experts invited, 60 formally agreed to participate. A high participation rate in the first and second rounds (92% and 83%, respectively) maintained the panel composition stable in terms of the characteristics considered (table 4). The proportion of physicians, psychologists and researchers was well balanced, with >70% of participants having a research and/or clinical experience of ≥15 years. At the first round, the experts clearly leaned towards the term 2 ’occupational burnout’. However, the definition statement proposed at the first round raised many comments. These comments mainly concerned six topics: (i) insufficient recognition of the ICD-11 definition, (ii) relevance of using the qualifier ’occupational’, (iii) terminology used for the concept definition, (iv) omission of symptoms other than exhaustion, (v) concern with the term ’exposure’, and (6) concern with the term ’problems at work’. The concerns about the ICD-11 definition further justified our decision to use SNOMED-CT’s terminology. Moreover, as a result of the experts’ comments, we accepted the suggestion to add the qualifier ’prolonged’ to the term ’exposure’ and to replace the term ’problem at work’ by ’work-related problems’. The revised definition submitted for the second round vote was as follows: *“In a worker, occupational burnout or occupational physical AND emotional exhaustion state is an exhaustion due to prolonged exposure to work-related problems”*. This definition received 82% of grades ≥7, and was consensually approved in the second round.

**Table 4:** Panel description and results obtained at the first and second rounds of Delphi on occupational burnout definition.[NA=not available.]

	Experts who agreed participating	Experts completing 1^st^ round	Experts completing 2^nd^ round

	N (%)	N (%)	N (%)
Number of participants	60 (100)	55 (92)	50 (83)
Gender			
Male	21 (35)	20 (36)	18 (36)
Female	39 (65)	35 (64)	32 (64)
Age (years)			
<30	1 (2)	1 (2)	1 (2)
30–44	21 (35)	19 (35)	18 (36)
45–60	27 (45)	24 (44)	21 (42)
>60	11 (18)	11 (20)	10 (20)
Highest education degree			
Bachelor	1 (2)	1 (2)	1 (2)
Master	7 (12)	6 (11)	6 (11)
MD	13 (22)	12 (22)	11 (22)
PhD	39 (65)	36 (65)	32 (64)
Field of education			
Medicine	37 (62)	36 (66)	34 (68)
Psychology	15 (25)	15 (27)	13 (26)
Life sciences	4 (7)	3 (5)	2 (4)
Other	4 (6)	1 (2)	1 (2)
Main occupation			
Occupational physician	20 (34)	18 (33)	18 (36)
Psychiatrist	5 (9)	4 (7)	4 (8)
General or other practitioner	3 (4)	3 (5)	3 (6)
Psychologist	12 (20)	12 (22)	10 (20)
Researcher	20 (33)	18 (33)	15 (30)
Length of occupational experience (years)			
<5	1 (2)	1 (2)	1 (2)
5–9	15 (25)	12 (22)	11 (22)
10–14	2 (3)	2 (4)	2 (4)
15–20	17 (28)	16 (29)	13 (26)
>20	25 (42)	24 (44)	23 (46)
Source of expertise on burnout			
Clinical practice	28 (47)	26 (47)	23 (46)
Research practice	46 (80)	42 (76)	38 (76)
Situation regarding the OMEGA-NET			
OMEGA-NET member	33 (55)	31 (56)	29 (58)
External participant	27 (45)	24 (44)	21 (42)
Term preferred for concept introduction	NA		
Term 1 ^a^	NA	17 (31)	12 (24)
Term 2 ^b^	NA	37 (69)	37 (76)
Degree of agreement on the concept definition	NA		
Mean ± Standard deviation	NA	5.9± 2.2	7.0± 1.6
Median	NA	6	7
Proportion of agreement (vote ≥7)	NA	23 (42)	41 (82)

a Term 1: ‘occupational physical AND emotional exhaustion state’.

b Term 2: ‘occupational burnout’.

## Discussion

The harmonized definition of occupational burnout that emerged from this study looks extraordinarily simple but responds to the fundamentals of definition formation. It is a conditional definition because the application of the concept introduced by the definition is conditional on specific circumstances, such as having an occupational activity, as indicated in the definition by the expression ’in a worker’. In general, a term introduced by a conditional definition cannot be replaced by its definiens in all contexts (7). Therefore, this term could also fit the ICD-11 hierarchy. Moreover, this definition is an operational definition (44, 45) as it suggests the use of a history taking procedure, assessing the problems at work, and a clinical examination to ascertain whether the patient suffers from physical and emotional exhaustion. In fact, the operationalization of an attribute is characterized by the indication of some operations (eg, clinical examination, history taking) that enables investigators to decide whether the attribute is present or absent (45).

The term ’work-related problems’ deserves discussion, as it was strongly debated among panelists. The challenge was to find a well-defined term that would cover most, if not all, work-related stressors or risk factors. In this respect, the concept ’problems at work’, defined in SNOMED-CT as an attribute of ’work and retirement-related problems’, was considered the best option. The concept ’problems at work’ has 12 hyponyms and involves 7 additional, more specific concepts, including ’discord in the workplace’, ’uncongenial work environment’, ’stressful work schedule’, and ’difficulty adjusting to work situation’ (table 3). Not all these examples are ’problems’. While it should be possible to extend the list of hyponyms under the concepts of ’problems at work’ or ’work-related problems’, it would be difficult to find a more inclusive and better-defined concept.

Regarding the meaning of the word ’problem’, we consulted three dictionaries to consider possible negative cultural perceptions associated with it. The Oxford English Dictionary (OED) defines a problem as “a difficult or demanding question; a matter or situation regarded as unwelcome, harmful, or wrong and needing to be overcome; a difficulty.” The Webster dictionary, defines a problem as “1a: a question raised for inquiry, consideration, or solution; b: a proposition in mathematics or physics stating something to be done; 2a: an intricate unsettled question; 2b: a source of perplexity, distress, or vexation; 2c: difficulty in understanding or accepting.” Finally, Cambridge Academic Content Dictionary, defines a problem as “a1: a situation, person, or thing that needs attention and needs to be dealt with or solved.” These three definitions, and in particular Webster’s definitions 2b and 2c seemed to fit pretty well the ’concerns’, ’constraints’, ’issues’ and ’situations’, mentioned by some of the experts, which could be all summarized using the term ’problems’. None of the other terms better fits our context as they are not well-defined terms within the SNOMED-CT and are subject to a wide interpretation according to the cited dictionaries. Therefore, the terms ’problems at work’ and ’work-related problems’ appeared to be the most convenient and clearest terms available. Indeed, they cover a large set of situations and have an extensible list of hyponyms, allowing for the introduction of new concepts corresponding to the additional work-related risk factors, if necessary. Finally, the term ’prolonged’ was added to the final definition as all the panelists agreed that it is important to specify the duration of exposure as part of necessary causal condition. The choice between the qualifiers ’chronic’ or ’prolonged’ to the term ’exposure’ was debated. According to SNOMED-CT, the terms ’prolonged’ and ’chronic’ are not synonyms. The term ’prolonged’ is defined as a qualifier value of duration and has no synonyms, while the term ’chronic’ is defined as a qualifier value of courses and has an acceptable synonym ’chronic course - prolonged duration’. In the OED, ’chronic’ is defined as “Lasting a long time, long-continued, lingering, inveterate; opposed to acute. Continuous or constant.” While ’prolonged’ is defined as “Of extended duration; protracted. Frequently with negative connotation. Extended, lengthened in space.” As exposure should not necessary be constant to result in a burnout, it appeared preferable to use the term ’prolonged’ to complete the definition. The timespan of ’prolonged’ still remains to be addressed. We believe that it would be possible, at least partially, in the systematic review of burnout predictors (in progress) and in the near future.

If accepted more generally, this definition may reduce the semantic chaos surrounding the concept of occupational burnout and improve medical research, treatment and prevention of this outcome. It may also clarify whether burnout should be classified as a disease (6, 46). In SNOMED-CT, ’burnout’ is classified under the clinical finding hierarchy, which only includes concepts that refer to diagnoses. Consequently, according to SNOMED-CT’s classification, ’burnout’ is a diagnosable disease, which is contradictory with the absence of a validated diagnostic standard. Before such a standard becomes available, professionals should be encouraged to use the most valid patient-reporting outcome measures of exhaustion. Although exhaustion constitutes the core component of occupational burnout, as highlighted in our definition, no fewer than 132 other possible symptoms (affective, cognitive, physical, behavioral, and motivational) have been mentioned in past literature reviews (21, 34). A thorough clinical examination of these symptoms would help define diagnostic criteria for occupational burnout.

### Study limitations

This study has at least three limitations. First, in order to select only studies of the highest quality with a documented definition of burnout in workers, our semantic research corpus excluded cross-sectional studies, studies published in other databases, and the grey literature, We identified 88 unique definitions in 248 studies. On the other hand, Rosenstein et al (1) reviewed 182 longitudinal and cross-sectional studies from five databases and identified at least 142 unique definitions. This suggests that the authors of the studies in Rosenstein et al’s review most likely used their own definitions, and we may have missed some that might be original. Nevertheless, we used the quantitative criteria in the semantic analysis based on the number of original definitions in the analytical sub-corpus. Hence, we can reasonably rule out a potential selection bias. Moreover, the semantic analysis, conducted prior to the consultation of SNOMED-CT, resulted in a similar definition as SNOMED-CT’s definition.

Second, our expert panel only represented countries that are part of OMEGA-NET. Therefore, we cannot speculate on the reproducibility of the experts’ selection and the representativeness of our panel in other countries. The use of a randomized sampling method for expert selection was not possible, but all EU-OSHA national focal points have a network to provide input to the EU-OSHA’s work and to disseminate products and information to national stakeholders. They presumably used this network to identify experts and assess their eligibility. External experts represented 45% of the panel and our statistical analysis showed that Delphi results were independent of the OMEGA-NET membership and other characteristics of the experts. Lastly, the size of our expert panel was not very large. This can affect the stability of the results. However, few Delphi studies on mental health had >50 experts (17).

Third, we conducted our literature review up to 2018. Although no new definition of burnout has been introduced in the scientific literature since then, several factor-analytic studies have recently concluded that burnout—and most notably, exhaustion—was reflective of a depressive condition (47, 48). Because our temporal limit was 2018, we did not incorporate these findings in our analyses. We note, however, that the harmonized definition of occupational burnout that emerged from the present study may be helpful in resolving the issue of burnout–depression overlap (49).

Future research should address the reproducibility of our results in a larger expert panel, representing more countries, and examine the utility of the formulated definition of burnout for researchers and practitioners.

## Protection of research participants

All participants provided formal consent for participation. Delphi panelists’ personal data used for the panel characterization were de-identified and destroyed after the study completion. Participation to the Delphi process was anonymous until the study completion and disclosed upon written consent for the authorship purpose.
